# DWI Positivity in Mild, Nondisabling Acute Cerebral Ischemia: Data From the PRISMS Trial

**DOI:** 10.1161/SVIN.124.001613

**Published:** 2025-01-17

**Authors:** Lily L. Wang, Pooja Khatri, Shyam Prabhakaran, Heidi Sucharew, Thomas A. Tomsick, Janice A. Carrozzella, Robert J. Stanton, Joseph Broderick, Dawn Kleindorfer, Steven R. Levine, Jose G. Romano, Jeffrey L. Saver, Sharon D. Yeatts, Achala Vagal

**Affiliations:** ^1^ Department of Radiology University of Cincinnati Cincinnati Ohio USA; ^2^ Department of Neurology University of Cincinnati Cincinnati Ohio USA; ^3^ Department of Neurology University of Chicago Chicago Illinois USA; ^4^ Department of Emergency Medicine University of Cincinnati Cincinnati Ohio USA; ^5^ Department of Neurology University of Michigan Ann Arbor Michigan USA; ^6^ Departments of Neurology and Emergency Medicine SUNY Downstate Health Sciences University Brooklyn New York USA; ^7^ Department of Neurology University of Miami Miller School of Medicine Miami Florida USA; ^8^ Department of Neurology and Comprehensive Stroke Center University of California Los Angeles USA; ^9^ Department of Public Health Sciences Medical University of South Carolina Charleston South Carolina USA

**Keywords:** cerebrovascular disease, DWI, MRI, stroke

## Abstract

**Background:**

In patients with acute cerebral ischemia (ACI) and mild deficit, acute infarction on magnetic resonance imaging (diffusion‐weighted imaging [DWI]‐positivity) is inconsistently detected. Our objective was to characterize the rate of detection in the first dedicated trial of a population with mild, nondisabling stroke and assess the detection and its association with baseline clinical and imaging characteristics. We hypothesized that white matter hyperintensity (WMH) would independently predispose patients to DWI‐positivity.

**Methods:**

The phase 3b, randomized, double‐blinded PRISMS (Potential of Alteplase for Ischemic Strokes with Mild Symptoms) trial compared administration of intravenous alteplase to aspirin for mild (National Institutes of Health Stroke Scale score 0–5), nondisabling stroke at ≤3 hours from onset. For the current, prespecified study, patients with a final diagnosis of neurovascular mimic were excluded. Central readers, blinded to the treatment arm, assessed day 2 magnetic resonance imaging for prior infarct, WMH burden (graded quantitatively [volume] and qualitatively [(Fazekas]), and presence of DWI‐positivity (primary outcome). Alteplase treatment, demographics, and clinical covariates were prespecified for adjustment in regression modeling.

**Results:**

Of 313 patients enrolled, 273 (87%) had a final diagnosis of ACI. Of 212 (77%) cases of ACI with magnetic resonance imaging, 109 (51%) had DWI positivity (median lesion volume 1.20 cc, interquartile range 0.57–2.50 cc). Univariately, the proportion with higher Fazekas grade (grade 2–3: 27% vs 41%; *P* = 0.02) and prior infarct (45% vs 27%, *P*<0.01) were associated with DWI positivity. WMH was not associated with DWI positivity in adjusted analysis (adjusted odds ratio [aOR]: 1.01, 95% CI: 0.99–1.03). The multivariable model also showed alteplase treatment inversely associated with DWI‐positivity (aOR: 0.48, 95% CI: 0.25–0.93).

**Conclusions:**

Among patients presenting with mild, nondisabling ACI, WMH is not associated with DWI‐positivity. A hypothesis emerges that alteplase treatment may reduce DWI positivity in mild, nondisabling ACI.

## Graphical abstract

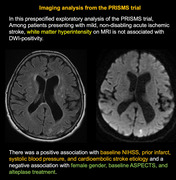

**Clinical Perspective**

- Among patients presenting with mild, nondisabling acute ischemic stroke, white matter hyperintensity on magnetic resonance imaging is not associated with diffusion‐weighted imaging‐positivity.
- We found that unexpectedly, alteplase treatment appeared to reduce diffusion‐weighted imaging‐positivity.

**Nonstandard Abbreviations and Acronyms**

ACI: acute cerebral ischemia
ASPECTS: Alberta Stroke Program Early CT Score
DWI: diffusion‐weighted imaging
NIHSS: National Institutes of Health Stroke Scale
PRISMS: Potential of Alteplase for Ischemic Strokes with Mild Symptoms
WMH: white matter hyperintensity

## Introduction

Mild strokes (defined as National Institutes of Health Stroke Scale [NIHSS] score ≤5 and “not clearly disabling” on presentation) account for ∼265 000 new ischemic strokes, ≈60% of all acute ischemic strokes, in the United States annually.^1^, ^2^, ^3^ Despite relatively mild deficits upon presentation, 20%–30% of such patients are disabled at 90 days post stroke.^3^, ^4^, ^5^ Accordingly, the PRISMS (Potential of Alteplase for Ischemic Strokes with Mild Symptoms) trial was designed to test the efficacy of intravenous (IV) alteplase at <3 hours from the onset of mild stroke.^4^ The trial ended prematurely due to recruitment challenges and was therefore underpowered for its primary analysis; however, the results did not show evidence of the potential benefit of alteplase and demonstrated the anticipated risk of intracranial hemorrhage. National guidelines were updated to recommend against treatment with alteplase, albeit with limited clinical evidence (Class III, Level B‐R), and the subsequent ARAMIS (Antiplatelet vs R‐tPA [recombinant tissue plasminogen activator] for Acute Mild Ischemic Stroke) and TEMPO‐2 (Randomized Controlled Trial of TNK‐tPA [tenecteplase‐tPA] Versus Standard of Care for Minor Ischemic Stroke With Proven Occlusion) trials confirmed these findings.^6^, ^7^ For nondisabling stroke with large vessel occlusion, endovascular therapy lacks randomized data at this time (Class IIb, Level C), with trials now underway.^8^, ^9^

In clinically moderate and severe strokes, neuroimaging nearly always demonstrates acute infarction and demonstrates arterial occlusions in up to 50% of patients. Mild strokes often have no or small acute infarcts and large vessel occlusion are less common. In fact, up to a third of clinically confirmed mild strokes do not have acute infarcts visible on magnetic resonance imaging (MRI) but still have significant disability.^10^ Although mild strokes with visible acute infarcts have more impairments compared to those without, 25% of patients without acute infarcts still experience poor quality of life.^11^

Baseline clinical and imaging characteristics, associated with increased vulnerability and tissue infarction, are not yet well characterized. Particularly, preexisting white matter hyperintensity (WMH) as a measure of diminished compensatory microcirculation, may lead to poor tolerance to ischemia and may influence these outcomes after mild stroke.^12^

Using the PRISMS trial data set, our prespecified study objective was to determine whether baseline clinical and imaging characteristics are associated with acute infarction after mild stroke. We hypothesized that the extent of WMH will be associated with the presence of infarction on MRI‐diffusion weighted imaging (DWI) after mild stroke, even after adjusting for clinical factors (age, vascular risk factors, baseline stroke severity) and alteplase treatment.

## Methods

### Data Set and Patients

The data that support the findings of this study are available from the corresponding author upon reasonable request. PRISMS was designed as a Phase 3b, double‐blind, randomized, placebo‐controlled trial of IV alteplase (with placebo aspirin) compared to placebo alteplase (with aspirin) at ≤3 hours. The primary outcome was 90‐day modified Rankin Scale score 0–1. The PRISMS inclusion criteria included mild, nondisabling ischemic stroke, age ≥ 18 years (no upper age limit), study treatment that could be initiated within 3 hours of last known well, and informed consent prior to initiation of any study‐specific treatment. Mild, nondisabling stroke was defined as a presenting NIHSS score of 0 to 5 with a deficit judged (by physicians/family/patient) at presentation to not prevent performance of basic activities of daily living or return to employment if applicable. Key exclusion criteria included hemorrhage or infarct greater than one third middle cerebral artery territory on baseline head computed tomography (CT), prestroke disability (modified Rankin Scale score ≥2) and standard contraindications to IV alteplase. The details of the trial have been published previously.^4^, ^13^

Although the study was terminated early due to recruitment, PRISMS is a rich data set of prospective patients with acute ischemic stroke with mild deficits. For the purposes of this analysis, our prespecified target population consisted of all patients who were assigned a final diagnosis of acute cerebral ischemia (ACI) and received day 2 MRI (22–48 hour MRI). All cases were categorized as either ACI or stroke mimic by site designation, and with central adjudication, in the primary trial. Specifically, if participants assigned ACI had neuroimaging evidence of new infarction, site investigators’ designations were accepted. The remainder were reviewed by blinded steering committee neurologists, and discordant assessments led to discussions between the site investigator and central reviewer to ensure that all relevant data were considered in the site's final determination.^4^

### Imaging Analysis

The study required follow‐up neuroimaging within 22 to 36 hours after intravenous study treatment bolus. Either computed tomography or MRI were permissible, and MRI was encouraged if consistent with institutional standard of care. All neuroimaging was centrally collected, and 2 independent central readers (experienced board‐certified neuroradiologists A.V. and T.T.) assessed the day 2 MRI studies. A consensus adjudication was used for discordant cases. The readers were blinded to treatment assignments and clinical outcomes.

The images were assessed for prior infarct, WMH burden, and presence of DWI‐positivity. The presence of DWI positivity was defined by hyperintensity on DWI with corresponding restricted diffusion on the apparent diffusion coefficient maps. The central readers followed Standards for Reporting Vascular Changes on Neuroimaging recommendations ^14^ for defining WMH and used both qualitative and quantitative measurements of WMH. The Fazekas scale was used for visual rating, using the fluid‐attenuated inversion recovery sequence.^15^ The quantitative, volumetric measurement of WMH on fluid‐attenuated inversion recovery was performed using MRICron ^16^ to create region of interest maps of supratentorial WMH by signal intensity thresholding followed by manual editing as necessary. Both confluent and focal WMH were quantified. Hyperintense lesions involving the subcortical gray matter, brainstem, U‐fibers, corpus callosum, internal capsule, anterior commissure, and chronic infarcts were not regarded as WMH.^14^ DWI was used for visual guidance to distinguish WMH from acute infarcts. Acute infarct volumes were also measured using manual lesion tracing using MRICron software.^17^ Patients who received CT angiography also had central reads for the presence of vessel occlusion. CT angiography positive was defined as the presence of vessel occlusion, which is defined as complete occlusion or hairline lumen.

### Statistical Analysis

Demographic and clinical characteristics were summarized with mean±SD or median and interquartile range for continuous data and count and percentage for categorical data. The primary outcome was the dichotomized presence or absence of acute infarction (DWI positive versus negative) on day 2 brain MRI according to the central reading. Among patients with day 2 MRI, demographic and clinical characteristics were compared between DWI group (positive versus negative) using chi‐square (or Fisher's exact) for categorical variables and Wilcoxon rank sum test for continuous variables. Similarly, demographic and clinical characteristics were compared between groups of patients with MRI and without MRI at day 2 using chi‐square (or Fisher's exact) for categorical variables and Wilcoxon rank sum test for continuous variables.

The association between the primary outcome and WMH volume was evaluated using logistic regression. Covariates in the model included study treatment that was received (IV alteplase vs placebo) and clinical variables of age, sex, baseline NIHSS score, prior infarct, baseline systolic blood pressure, baseline Alberta Stroke Program Early CT Score (ASPECTS), cardioembolic stroke etiology, smoking status, and baseline glucose level. A WMH by treatment group interaction term was considered in the model and removed if not statistically significant at *P*<0.10. For continuous variables in the model, the linearity assumption with the log of the odds was checked by including restricted cubic splines with 3 knots for each continuous variable. A Wald test was used to jointly test the spline parameters, which were removed from the model if not statistically significant at *P*<0.10.

## Results

Of the total 313 patients enrolled in PRISMS, 273 (87%) had a final diagnosis of ACI. A total of 212 patients out of 273 (77%) had day 2 MRI scans available. Patients who received an MRI were younger, more likely to be female, and less likely to have atrial fibrillation. as compared to those who did not get an MRI. (Supplementary Table 1). Of the 212 patients,109 patients (51%) had DWI positivity and the median acute infarct volume was 1.20 cc (interquartile range 0.57–2.50 cc).

Table 1 shows the demographic characteristics according to DWI positivity. Participants who had MRIs with no evidence of positivity on DWI imaging were more likely to be younger (58 vs 63, *P*<0.01), treated with t‐PA than aspirin (57% vs 44%; *P* = 0.05), female than male (54% vs 42%; *P* = 0.02), higher ASPECTS scores (*P* = 0.01), and higher Fazekas scores of white matter disease (p = 0.01).

**Table 1:** Demographics and Clinical Factors by Diffusion Positivity

	Acute cerebral ischemia (N = 273)	With MRI (N = 212)	DWI + (N = 109)	DWI ‐ (n = 103)	*P* value
Age, y, mean±SD	62 (12) [n = 267]	61 (12) [n = 211]	63 (13) [n = 108]	58 (11)	<0.01
Female, n (%)	122 (45%)	102 (48%)	44 (40%)	58 (56%)	0.02
Race, n (%)					0.57
White	208 (76%)	159 (75%)	82 (75%)	77 (75%)	
Black	57 (21%)	46 (22%)	25 (23%)	21 (20%)	
Other	6 (2%)	5 (2%)	1 (1%)	4 (4%)	
Missing	2 (1%)	2 (1%)	1 (1%)	1 (1%)	
Baseline National Institutes of Health Stroke Scale score, n (%)					0.44
0	10 (4%)	8 (4%)	2 (2%)	6 (6%)	
1	79 (29%)	60 (28%)	27 (25%)	33 (32%)	
2	92 (34%)	66 (31%)	36 (33%)	30 (29%)	
3	50 (18%)	43 (20%)	23 (21%)	20 (19%)	
4	32 (12%)	28 (13%)	16 (15%)	12 (12%)	
5	10 (4%)	7 (3%)	5 (5%)	2 (2%)	
Mean±SD	2.2 (1.2)	2.2 (1.2)	2.4 (1.2)	2.0 (1.2)	0.07
Prior infarct, n (%)	99 (36%)	77 (36%)	49 (45%)	28 (27%)	0.01
History of diabetes	95 (35%)	73 (34%)	35 (32%)	38 (37%)	0.46
History of hypertension	223 (82%)	170 (81%)	91 (83%)	79 (77%)	0.22
History of atrial fibrillation	39 (14%)	20 (9%)	12 (11%)	8 (8%)	0.42
Smoking status, n (%)					0.87
Current	66 (24%)	54 (25%)	29 (27%)	25 (24%)	
Former	86 (32%)	67 (32%)	35 (32%)	32 (31%)	
Never	121 (44%)	91 (43%)	45 (41%)	46 (45%)	
Glucose level mmol/L, mean±SD	7.7 (3.7) [n = 270]	7.7 (3.9) [n = 209]	8.0 (4.1) [n = 108]	7.5 (3.8) [n = 101]	0.11
Systolic blood pressure, mm Hg, mean±SD	148 (20.9) [n = 263]	147 (21.3) [n = 204]	150 (20) [n = 103]	144 (22) [n = 101]	0.03
Alberta Stroke Program Early CT Score, median (range)	10 (7‐10)	10 (7‐10)	10 (7‐10)	10 (9‐10)	0.01
Tissue plasminogen activator received	136 (50%)	107 (50%)	48 (44%)	59 (57%)	0.05
Last known well to treatment hour, n (%)					0.79
0–2 h	56 (21%)	43 (20%)	21 (19%)	22 (21%)	
>2–3 h	210 (77%)	164 (77%)	86 (79%)	78 (76%)	
>3 h	7 (3%)	5 (2%)	2 (2%)	3 (3%)	
Mean±SD	2.5 (0.5)	2.5 (0.5)	2.5 (0.5)	2.5 (0.5)	0.71
Stroke etiology					<0.01
Cardioembolic	37 (14%)	25 (12%)	22 (20%)	3 (3%)	
Large‐artery atherosclerosis	30 (11%)	22 (10%)	11 (10%)	11 (11%)	
Small‐artery occlusion (lacune)	100 (37%)	79 (37%)	38 (35%)	41 (40%)	
Other determined etiology	20 (7%)	16 (8%)	7 (6%)	9 (9%)	
Stroke of undetermined etiology	86 (32%)	70 (33%)	31 (28%)	39 (38%)	
Fazekas					0.01
0		89 (42%)	36 (33%)	53 (51%)	
1		78 (37%)	43 (39%)	35 (34%)	
2		37 (17%)	23 (21%)	14 (14%)	
3		8 (4%)	7 (6%)	1 (1%)	
White matter hyperintensities volume, median (interquartile range)		5.8 (0, 23.6)	11.7 (0, 27.7)	0 (0, 13.9)	<0.01
90‐d mRS score 0 or 1, nonimputed			79/102 (77%)	79/95 (83%)	0.32
90‐d mRS score 0 or 1 (using 30 d for missing and out of window)			83/108 (77%)	84/101 (83%)	0.25

*P* values comparing DWI + and ‐ groups from chi‐square test or Fisher's exact test for categorical variables and from Wilcoxon rank sum test for continuous variables

*P* value from chi‐square test or Fisher's exact test for categorical variables and from Wilcoxon rank sum test for continuous variables.

DWI indicates diffusion‐weighted imaging; and mRS, modified Rankin Scale.

Table 2 shows the results of the multivariable logistic model for the outcome of DWI positivity. There was no sufficient evidence of a WMH by treatment group interaction (*P* = 0.26) and it was removed from the model. The linearity of the continuous variables was checked, and the model did not improve with restricted cubic splines. Therefore, linear terms are included in the model. Unadjusted, WMH was associated with DWI positivity (odds ratio [OR], 1.02; 95% CI, 1.01–1.04). However, WMH was not associated with DWI positivity in adjusted analysis (OR, 1.01; 95% CI, 0.99–1.03). DWI positivity was negatively associated with female sex (OR, 0.47), baseline ASPECTS (OR, 0.09), and alteplase treatment (OR, 0.48). DWI positivity was associated with baseline NIHSS score (OR, 1.35), prior infarct (OR, 2.39), systolic blood pressure (OR, 1.02), and cardioembolic stroke etiology (OR, 10.08).

**Table 2:** Multivariable Logistic Regression Model of DWI Positivity on MRI

	OR (95% CI)
*Unadjusted*:	
WMH volume, per 1 cc	1.02 (1.01–1.04)
*Adjusted*:	
WMH volume, per 1 cc	1.01 (0.99–1.03)
Age, per 1 y	1.01 (0.98–1.04)
Female	0.47 (0.24–0.91)
Baseline National Institutes of Health Stroke Scale score, per 1 point	1.35 (1.01–1.79)
Prior Infarct	2.39 (1.19–4.80)
Systolic blood pressure, per 1 mmHg	1.02 (1.00–1.04)
Baseline Alberta Stroke Program Early CT Score, per 1 point	0.09 (0.01–0.95)
Alteplase treated	0.48 (0.25–0.93)
Cardioembolic stroke etiology	10.08 (2.60–39.10)
Smoking status	
Current	0.88 (0.37–2.08)
Former	0.87 (0.40–1.88)
Never	Reference
Baseline glucose level, per 1 mmol/L	1.03 (0.95–1.12)

DWI indicates diffusion‐weighted imaging; MRI, magnetic resonance imaging; OR, odds ratio; and WMH, white matter hyperintensities.

Good outcome (90‐day modified Rankin Scale score 0–1) was seen in 83/106 (78%) of alteplase‐treated and 84/103 (82%) of aspirin‐treated patients, consistent with the primary intention‐to‐treat analysis in the full cohort. Of the 5 patients with symptomatic intracranial hemorrhage in PRISMS, all were alteplase treated.

## Discussion

In this prespecified exploratory analysis of the PRISMS trial, we did not demonstrate an association between WMH and DWI positivity as hypothesized. We, however, showed a positive association with baseline NIHSS score, prior infarct, systolic blood pressure, and cardioembolic stroke etiology and a negative association with female gender, baseline ASPECTS, and alteplase treatment. The lack of an association between WMH burden and DWI positivity is in contrast to prior studies that have shown that WMH burden predicts ischemic stroke risk.^18^ A recent study also showed that WMH burden differs by stroke types and those with small artery occlusion had higher WMH volume compared to other stroke subtypes.^19^ The location, in addition to the volume of the WMH, may be important and differ among subgroups of patients.^20^, ^21^ It has been proposed that the capacity of the brain to either avert tissue injury (no visible infarct) or minimize its extent (small volume infarct) may be estimated by the degree of chronic microvascular ischemia. It is possible that our sample size was too small or too homogeneous with its predominance of stroke attributed to small vessel disease.

In our analysis, only approximately half of the patients developed an MRI‐visible infarction, with very small infarct volumes overall. This reinforces the fact that, in patients with mild stroke, the presence and extent of infarct visualized on MRI is indeed highly variable. The concept of a DWI‐negative ischemic event has been well described ^2^, ^22^, ^23^ and may be related to 2 reasons. First, false‐negative imaging depends on spatial resolution and some ischemia may be below the ability of MRI detection. Second, infarction or transient ischemic attack may be averted in the setting of rapid reperfusion of ischemic tissue, either spontaneously or due to administration.^24^, ^25^, ^26^ In addition, small symptomatic infarct lesions may be missed on MRI in brain regions masked by artifacts, particularly in the brainstem, and between slice gaps.^27^, ^28^, ^29^

We found that unexpectedly, alteplase treatment appeared to reduce DWI positivity, a hypothesis‐generating finding. There was, however, no difference in the 3‐month outcome. This may be due to the small sample size or the timing of the outcome measure. Acute reperfusion therapy (IV alteplase or endovascular therapy) is associated with a reduction in final infarct volume in moderate and severe strokes and may be similarly associated with absent or smaller final infarct volume in mild strokes.^30^, ^31^ Whether thrombolysis has a biological effect and reduces or eliminates final infarct volume in patients with mild stroke is a recommended area of future investigation.

Our study has several important limitations. Imaging studies in PRISMS were all performed as part of standard of care and hence used heterogenous MR and CT angiography protocols performed routinely at sites. However, all MRI studies had minimum sequences (fluid‐attenuated inversion recovery, DWI, apparent diffusion coefficient, T1‐weighted, and T2‐weighted), allowing for generalizable findings. Also, due to standard of care imaging in the study, advanced imaging such as perfusion and vascular imaging to assess for evidence of hemodynamic alterations, particularly in the DWI negative cases, were unavailable consistently. Another limitation is the single time point of measurement of DWI positivity; it is possible that later imaging would have shown infarct growth with subsequent visibility on DWI. However, among transient ischemic attack and minor strokes in the prospective CATCH (Coronary Atherosclerosis T1‐Weighted Characterization) study, such radiographic progression occurred in only 5.5% of patients with a median growth of 3.7 mL.^32^

In conclusion, we found that among patients presenting with mild, nondisabling ACI, the presence of WMH is not associated with DWI‐positivity. A hypothesis emerges that alteplase treatment for mild, nondisabling deficits may reduce the risk of developing DWI+ MRI lesions.

## Sources of Funding

Genentech Inc funded the trial.

## Disclosures

Lily L. Wang: Central reader for National Institutes of Health (NIH)‐funded trials FASTEST, SISTER, and VERIFY. Cerenouvs/Johnson & Johnson (ENDOLOW trial) central reader.

Pooja Khatri: Research grant from Cerenovus/Johnson & Johnson (ENDOLOW trial); consulting fees for scientific advisory roles from Lumosa, Basking Biosciences and Shionogi; drug and assays from Translation Sciences (for NIH‐funded SISTER trial); and royalties from UpToDate (online publication royalties). She also received research effort funding from Genentech (PRISMS trial) more than 5 years ago.

Janice A. Carrozzella: Salary support.

Jeffrey L. Saver: Reports consulting fees for advising on rigorous and safe clinical trial design and conduct from Abbott, Acticor, Aeromics, Amgen, Argenica, Astrocyte, Bayer, Biogen, Boehringer Ingelheim, BrainsGate, BrainQ, CSL Behring, Filterlex, Genentech, Johnson&Johnson, MindRhythm, Medtronic, NeuroMerit, Neuronics, Novo Nordisk, Occlutech, Phenox, Phillips, QuantalX, Rapid Medical, Roche, and Stream Biomedical.

Sharon D. Yeatts: Grant funding from NIH; fees to institution for DSMB service (Bard and Emory University), personal fees for editorial service (American Heart Association/American Stroke Association) and Steering Committee service (Genentech)

Robert J. Stanton: R01NS3078

Joseph P. Broderick: Reports receiving multiple research awards from the National Institutes of Neurological Disorders and Stroke (NINDS); study medication and monetary support from Novo Nordisk for temperature monitoring and after‐hour enrollment for the ongoing NINDS‐funded FASTEST trial; and monies for educational and research funds for the University of Cincinnati Department of Neurology and Rehabilitation Medicine from Genentech (for his role on the Executive Committee of the TIMELESS trial) and for consulting for Roche, BrainsGate Ltd, Basking Biosciences, and the Pharmacy and Therapeutics Committee of Kroger Prescriptions Plans, Inc, as a voting member of the committee.

Jose G. Romano: Research funding from the National Institute of Neurological Disorders and Stroke, the National Institute on Minority Health and Health Disparities, and the State of Florida Department of Health.

Heidi Sucharew: none

Achala Vagal: NIH/NINDS NS103824, NIH NINDS/NIA NS117643, NIH/NINDS NS100417, NIH/NINDS NS 069763, NIH/NINDS NS120493, NIH/NINDS U01 NS120910, NIH/NINDS U01NS100699, NIH/NINDS U01NS110772, NIH/NINDS U01NS117450, PI Imaging Core Lab ENDOLOW Trial, Cerenovus, Consultant, Viz.ai,Inc, Consultant, Cerebra AI

## Supporting information




**Supplementary Table 1**. Demographics and clinical factors by MRI imaging status
