# Supplementary material for: DWI Positivity in Mild, Nondisabling Acute Cerebral Ischemia: Data From the PRISMS Trial
Source: Stroke Vasc Interv Neurol. 2025 Jan 17;5(2):e001613. doi: 10.1161/SVIN.124.001613 (PMC12671632; doi:10.1161/SVIN.124.001613)
Supplement: Supplementary file 1 — Supplementary Table 1. Demographics and clinical factors by MRI imaging status [file SVI2-5-e001613-s001.pdf]

SUPPLEMENTAL MATERIALS

DWI Positivity in Mild, Nondisabling Acute Cerebral Ischemia: Data from the PRISMS Trial

Lily L. Wang, Pooja Khatri, Shyam Prabhakaran, Heidi Sucharew, Thomas A. Tomsick, Janice A. Carrozzella, Robert J. Stanton, Joseph Broderick, Dawn Kleindorfer, Steven R. Levine, Jose G. Romano, Jeffrey L. Saver, Sharon D. Yeatts, Achala Vagal

Author affiliations

LW – Department of Radiology, University of Cincinnati

PK – Department of Neurology, University of Cincinnati

HS – Department of Emergency Medicine, University of Cincinnati

TT - Department of Radiology, University of Cincinnati

JAC – Department of Radiology, University of Cincinnati

RJS - Department of Neurology, University of Cincinnati

JB - Department of Neurology, University of Cincinnati

DK – Department of Neurology, University of Michigan

SRL – Departments of Neurology and Emergency Medicine, SUNY Downstate Health Sciences University

JGR Department of Neurology, University of Miami Miller School of Medicine, Miami, FL

JLS – Department of Neurology and Comprehensive Stroke Center, University of California, Los Angeles

SDY – Department of Public Health Sciences, Medical University of South Carolina, Charleston, SC

AV - Department of Radiology, University of Cincinnati

Supplementary Table 1. Demographics and clinical factors by MRI imaging status

	Enrolled patients<br><br>(N=313)	With MRI<br><br>excluding stroke mimics<br><br>(N=212)	No MRI<br><br>excluding stroke mimics<br><br>(N=61)	p-value
Age, mean (SD)	61 (13)	61 (12)	67 (12)	<0.01
Female, n (%)	144 (46%)	102 (48%)	20 (33%)	0.03
Race, n (%)				0.46
White	243 (78%)	159 (75%)	49 (80%)	
Black	62 (20%)	46 (22%)	11 (18%)	
Other	6 (2%)	5 (2%)	1 (2%)	
Missing	2 (1%)	2 (1%)	0	
Baseline NIHSS, n (%)				0.27
0	14 (4%)	8 (4%)	2 (3%)	
1	88 (28%)	60 (28%)	19 (31%)	
2	102 (33%)	66 (31%)	26 (43%)	
3	62 (20%)	43 (20%)	7 (11%)	
4	37 (12%)	28 (13%)	4 (7%)	
5	10 (3%)	7 (3%)	3 (5%)	
Mean (SD)	2.2 (1.2)	2.2 (1.2)	2.0 (1.1)	0.23
Prior Infarct, n (%)	101 (32%)	77 (36%)	22 (36%)	0.97
History of Diabetes	101 (32%)	73 (34%)	22 (36%)	0.81
History of Hypertension	250 (80%)	170 (81%)	53 (87%)	0.23
History of Atrial Fibrillation	40 (13%)	20 (9%)	19 (31%)	<0.01
Smoking status, n (%)				0.58
Current	77 (25%)	54 (25%)	12 (20%)	
Former	94 (30%)	67 (32%)	19 (31%)	
Never	141 (45%)	91 (43%)	30 (49%)	
Missing	1 (0.3%)			
Glucose level mmol/L, mean (SD)	7.6 (3.8)	7.7 (3.9)	7.4 (2.8)	0.86
Systolic blood pressure mmHg, mean (SD)	146.9 (20.6)	147.2 (21.3)	149.1 (19.3)	0.58
Baseline ASPECTS, median (range)	10 (7-10)	10 (7-10)	10 (7-10)	0.54
tPA received		107 (50%)	29 (48%)	0.69
Treatment group assigned, n (%)				0.59
Intravenous Alteplase + Oral Placebo	156 (50%)	109 (51%)	29 (48%)	
Intravenous Placebo + Oral Asprin	157 (50%)	103 (49%)	32 (52%)	
Last known well to treatment hour, n (%)				0.77
0-2 hours	61 (19%)	43 (20%)	13 (21%)	
>2 – 3 hours	245 (78%)	164 (77%)	46 (75%)	
>3 hours	7 (2%)	5 (2%)	2 (3%)	
Mean (SD)	2.5 (0.5)	2.5 (0.5)		0.26
Stroke Etiology				0.50
Cardioembolic	37 (12%)	25 (12%)	12 (20%)	
Large-artery atherosclerosis	30 (10%)	22 (10%)	8 (13%)	
Small-artery occlusion (lacune)	100 (32%)	79 (37%)	21 (34%)	
Other determined etiology	20 (6%)	16 (8%)	4 (7%)	
Stroke of undetermined etiology	86 (27%)	70 (33%)	16 (26%)	
Missing – stroke mimics	40 (13%)			

P-value comparing infarct versus no infarct from chi-square test, or Fisher's exact test for categorical variables and from Wilcoxon rank sum test for continuous variables.
